# Safety in spinal surgery—Empowering clinicians to report concerns in motor function

**DOI:** 10.1111/jan.16399

**Published:** 2024-08-20

**Authors:** Jennifer Burrows, Eniola Dada, Brjan Betzler, Louise Strickland, Gerard Mawhinney

**Affiliations:** ^1^ Oxford Spine Service Oxford University Hospitals Oxford UK; ^2^ Department of Orthopaedic Surgery Tan Tock Seng Hospital Singapore Singapore; ^3^ Nuffield Department of Orthopaedics Rheumatology and Musculoskeletal Sciences, University of Oxford Oxford UK; ^4^ Nuffield Department of Clinical Neurosciences University of Oxford Oxford UK

**Keywords:** multiprofessional education, neurology, neurosurgery, nurse education, nursing assessment, nursing observations, orthopaedics, patient safety, quality, quality of care

## Abstract

**Aims:**

Timely identification of neurological deterioration in patients with spinal disorders, through spinal motor assessment, is paramount in achieving early intervention to reduce the risk of permanent deficits. This project was initiated to meet the requirement for safe, timely spinal motor assessment through establishing and addressing clinician's educational needs.

**Design:**

Mixed methods study conducted through online survey and concurrent focus groups June 2022–April 2023.

**Methods:**

Pre‐intervention online survey and focus groups identified insufficient provision of education targeted at identifying changes in motor function and as a result, clinicians lacked confidence and competence in completing assessments and caring for patients with spinal disorders. An e‐learning package was created and shared widely along with additional interventions to support assessment completion. To establish the success of the project a post‐intervention online survey was distributed.

**Results:**

Survey respondents reported that the e‐learning package has influenced their practice to either some extent or to a great extent with 91% reporting increased confidence in completing a spinal motor assessment. Post‐intervention results also demonstrated an increase in confidence in caring for spinal surgery patients.

**Conclusion:**

Through engaging with clinicians to establish and address educational needs, this quality improvement project has successfully increased competence and confidence in this area of spinal care.

**Implications for the Profession and/or Patient Care:**

This study highlights the importance of targeted education to ensure that clinicians are appropriately skilled to identify neurological deterioration and demonstrates the effectiveness of digital education in providing this.

**Impact:**

This study addressed concerns around timely identification of deterioration of spinal patients. Study findings were the success in utilizing digital education to increase clinician's confidence and competence and thus enhance patient safety. This research will have an impact on clinical areas caring for patients with spinal disorders.

**Reporting Method:**

SQUIRE guidelines.

**Patient or Public Contribution:**

No patient or public contribution.

## 1 INTRODUCTION

Timely spinal motor assessment (SMA) is an essential component of care of the spinal patient (The National Institute for Health and Care Excellence (NICE), 2016). Completing this assessment can identify recovery of function, and therefore success of intervention, but is also an important factor in identifying neurological deterioration (Byrne et al., 2000). Neurological deterioration of patients with spinal disorders is identified through loss or reduction in motor power or sensation below the level of injury and is indicative of suboptimal cord function (Buchanan et al., 2016). Completing and documenting neurological observations prior to and following spinal surgery is, therefore, essential in identifying these changes early in order for prompt investigation and appropriate intervention to take place. Delayed intervention in these circumstances can result in irreversible reduction or loss of motor power or sensation, prolonged hospital stays, poor recovery or even death (Badhiwala et al., 2021; Vaccaro et al., 2004; Willhuber et al., 2019).

## 2 BACKGROUND

Spinal motor assessment refers to the physical assessment of a person's myotomes (C5‐T1 and L2‐S1), utilizing the Medical Research Council (MRC) Motor Grading Scale to grade the power. Myotomes are defined as groups of muscles supplied by a single nerve root (Byrne et al., 2000); therefore, assessment of these myotomes can be utilized to gain information about the level in the spine where a lesion may be present (Byrne et al., 2000) and can identify changes in motor power of a person with a spinal disorder—both improvements as well as deterioration. In the presence of spinal cord injury the International Standards for Neurological Classification of Spinal Cord Injury produced by the American Spinal Injuries Association (ASIA) in conjunction with the International Spinal Cord Society (ISCoS) gives clear guidance on how to complete a motor power assessment using a standardized method that is proven to be reproducible amongst clinicians (Krisa et al., 2013; Savic et al., 2007). This assessment can be safely completed by any clinician who has been appropriately trained in both the completion of the assessment but also the ability to identify changes and an understanding of the urgency of seeking medical help when indicated. This assessment is taught routinely to doctors and physiotherapists as part of their undergraduate training. Other clinicians are more likely to be taught this as part of role‐specific training when working in an environment in which this is required. It may be taught by anyone who themselves has been taught to complete this assessment and competence is rarely formally assessed in the clinical setting but more often self‐declared.

**FIGURE 1:**
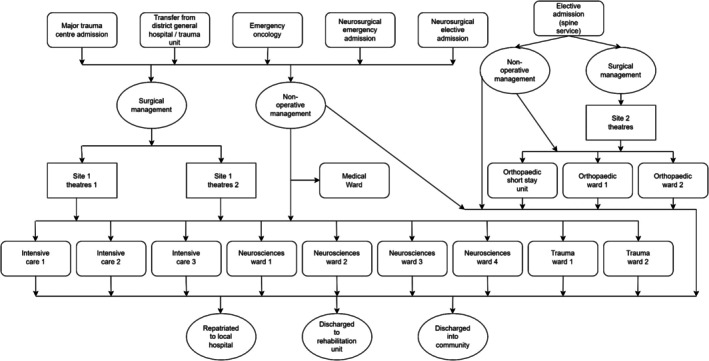
Inpatient journey for patients with spinal disorders.

Within the trust in which the study took place patients with spinal disorders could be routinely cared for in one of over 11 different ward areas (Figure. 1) with the potential to be cared for in additional ward environments as dictated by other injuries, comorbidities or bed capacity. Concerns were raised via the trust incident reporting system over specific cases of neurological deterioration with no clear record documenting when this was initially presented and how it was escalated. These circumstances led to the project team initiating informal discussion with clinicians routinely caring for these patients. Through these conversations, clinicians reported a lack of confidence and competence in their assessment and knowledge of identifying deterioration of patients with spinal disorders along with a lack of clarity on how to escalate concerns.

Prior to this project taking place all training within the trust related to caring for patients with spinal disorders was department‐specific and led by the practice development leads within each of the clinical areas. This training varied between departments but generally involved face‐to‐face classroom teaching which gave an overview of spinal anatomy and physiology, key aspects of caring for this patient group and an outline of signs of deterioration in patients with spinal disorders. Clinical education teams are also able to provide additional teaching and support within clinical areas where a need is identified. In no areas of this trust did standard teaching on the care of spinal patients cover the specifics of how to complete and document a full spinal motor assessment in order to identify deterioration.

The aim of this study was to promote the safety of patients with spinal disorders through ensuring clinicians were able to identify and escalate neurological deterioration in a timely manner through safe, timely spinal motor assessment.

The objectives of the project were as follows:

1. To assess the confidence and competence across the trust of clinicians caring for patients with spinal disorders in completing a spinal motor assessment
2. To identify gaps in knowledge in identifying and understanding neurological deterioration
3. To establish whether these gaps could be addressed through current training provision
4. Identify any other factors that may pose challenges to clinicians in identifying and escalating neurological deterioration
5. Address the factors identified in order to promote patient safety

In order to achieve these objectives, it was vital to identify the educational needs of clinicians caring for patients with spinal disorders. This preliminary work would guide interventions to be put in place to ensure appropriate competence and confidence in identifying neurological deterioration early, thereby reducing the risk of preventable harm.

### 2.1 The study

This study utilized a mixed methods design through use of qualitative and quantitative components with the response to the research questions supported by integrating information generated from both of these components (Regnault et al., 2018). Quantitative data were obtained through online surveys with this collection method also obtaining qualitative data but for the purposes of the study design, the qualitative data are in reference to the focus group data.

This study utilized the SQUIRE 2.0 guidelines as a framework for reporting this work.

## 3 METHODOLOGY

The study took place between June 2022 and April 2023 within a large acute NHS trust that serves as a major trauma centre and supra‐regional referral unit for spinal surgery and neurosurgery. Over 2500 patients with spinal disorders are admitted to the trust each year under the care of either the Spine Service or Neurosurgery.

The core project team included:

- Spinal Nurse Consultant—Identified requirement for project, recruited project leads, oversaw project leads, supported project roll‐out, supported project write‐up.
- Deputy Director of Nursing and Midwifery Research and Innovation (Clinical Academic Nurse Researcher and Trainee Surgical Care Practitioner within the spine service during project initiation and roll out.)—Sourced funding, recruited project leads, oversaw project leads, supported project roll‐out, supported project write‐up.
- Project Physiotherapy Lead—Collected and analysed data, created and implemented interventions, led project write‐up.
- Project Nurse Lead—Collected and analysed data, created and implemented interventions.
- Medical Student—Focus group data analysis, assisted in creation of interventions

Initially, trust‐wide multidisciplinary stakeholders were identified in order to gain insight into practice across all areas of the trust caring for patients with spinal disorders. Stakeholder meetings were held to facilitate conversation around the perceived challenges within the clinical areas and how best to engage clinicians prior to finalizing the project design. To allow for opinion from multiple professions, stakeholders included consultant surgeons from both the Spine Service and Neurosurgery as well as leads from intensive care units, the emergency department, departmental matrons, nursing practice development leads and therapy leads. Members of the digital informatics and electronic records team were also included.

### 3.1 Inclusion/exclusion criteria for the online surveys and focus groups

#### 3.1.1 Inclusion criteria

- Clinical staff.
- Employed by the trust.
- Working in patient‐facing roles.
- Working on any of the trust sites.

#### 3.1.2 Exclusion criteria

- Does not have a trust email address.

### 3.2 Interventions

The interventions actioned to address the outcomes of the survey are detailed below with respect to education, additional resources and documentation. These interventions were implemented in a way that was cost neutral to the NHS through utilization of in‐house services and resources. Funding was sought externally to cover staffing and production of the resources for which a cost was attached.

#### 3.2.1 Education

In order to provide education that was available trust wide and accessible to all health professionals, a digital training package was created. This was developed by the project leads in partnership with the trust team responsible for the online learning platform. The content was evidence based with additional expert opinion and contribution from the spinal consultant team. Feedback was sought from nursing staff caring for patients with spinal disorders to ensure that the information was presented in a way that was optimal for their learning. This training package covered:

- Spinal anatomy and physiology and how this links to patient presentation in terms of sensation and motor power.
- Pathophysiology of deterioration in patients with spinal disorders/following spinal surgery and how this may present clinically.
- How to complete a spinal motor assessment in order to identify changes in or loss of motor power.

Baseline data also identified a desire for face‐to‐face training with 73% of respondents to the questionnaire feeling this would be of benefit. Due to the short‐term funding for the project, this was something that the project team would be unable to implement long term. To enable face‐to‐face support to continue long term; volunteers were identified to act as Spinal Motor Assessment Champions for their clinical area. These champions were given additional education through funding to complete the American Spinal Injuries Association (ASIA) International Standards Training e‐Programme (American Spinal Injuries Association, 2023) as well as the option to attend face‐to‐face training with a member of the project team. By utilizing a ‘Train the Trainer’ model; these champions can use their additional knowledge and skills to act as in‐house trainers to support their colleagues in their learning and completion of patient assessments.

#### 3.2.2 Additional resources

**FIGURE 2:**
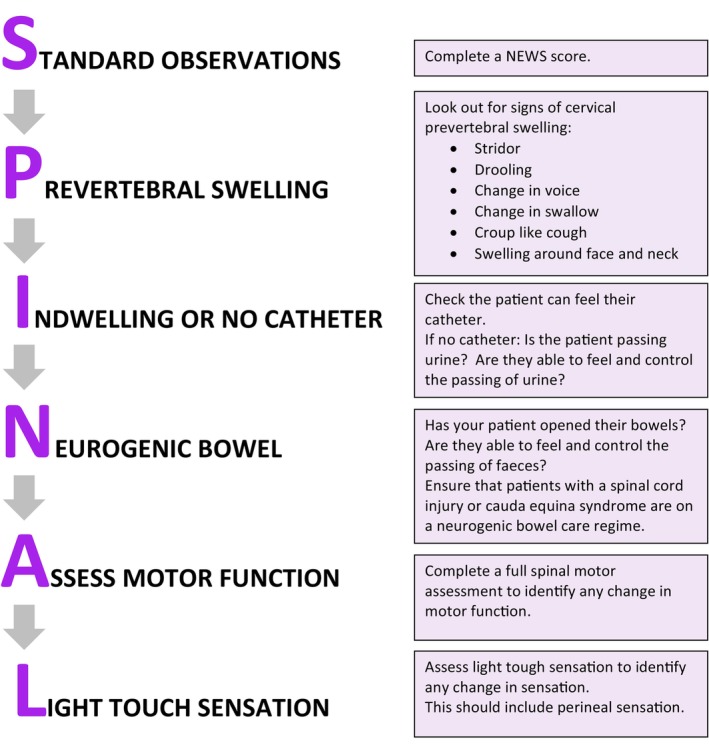
Spinal assessment flow chart.

Baseline data demonstrated a desire for additional resources to support spinal motor assessment completion with 95% of questionnaire respondents feeling that this would be of benefit in some format. When given examples of types of resources, there was no clear format that staff identified as being of more benefit than others; therefore, multiple options of resources were created:

- A video refresher link.
- A written guide to completion of a spinal motor assessment linked to the Electronic Patient Records (EPR).
- Flow chart posters for completion of a comprehensive assessment of a patient with a spinal disorder (Figure. 2).
- Ward posters to summarize how to complete a spinal motor assessment.
- Prompt cards for identifying myotomes and Medical Research Council Muscle grading that could be attached to lanyards.
- Escalation flow chart posters to clearly identify who to contact in the event of deterioration.

#### 3.2.3 Documentation

Fifty‐eight per cent of survey respondents felt that an electronic documentation template would be useful with focus group participants identifying a lack of knowledge on how to clearly document a motor power assessment. Buchanan et al. (2016) recommend the use of a standardized proforma and documentation chart as the minimum standard for the assessment of post‐operative neurological observations in spinal surgery.

The existing semi‐digital Spinal Surgery Assessment band within EPR was reviewed in terms of its ease of use and whether it was comprehensive in the aspects of spine care requiring clear, consistent documentation. It was identified that within this band, there was not the option to document a full motor assessment, signs of cervical prevertebral swelling or changes in bladder or bowel function.

In order to support staff in completing clear documentation of their assessment, a template was created that guides the user through a spinal motor assessment producing a clear record of the patient's motor power allowing easy comparison and, therefore, identification of deterioration. This includes a link to the written spinal motor assessment guidance document. This template also encompasses assessment of cervical prevertebral swelling, light touch sensory assessment and documentation of bladder and bowel function.

### 3.3 Data collection

Prior to the intervention, an online survey (Data S1) was designed by the project leads and reviewed by the rest of the project team to agree on clear, focused questions that would provide the required information. These questions were generated through concerns raised via stakeholder meetings as well as from informal discussions between the project team and clinicians caring for patients with spinal disorders. Due to time constraints, this was not piloted. This survey allowed for both quantitative and qualitative data collection and was distributed via trust wide communications and through divisional leads to allow for clinicians from all areas of the trust to contribute. Responding to the survey was entirely voluntary and relied on clinicians accessing the link via the emails disseminated.

In addition to the online survey, four focus groups were run concurrently with a total of 34 participants from Neurosciences wards, Neuro Intensive Care and the Orthopaedic Centre. Staff from Critical Care and Trauma were also invited to attend but were unable to due to staffing levels. These focus groups gave clinicians the opportunity to give further detail of their current practice, their training needs and the challenges they felt in caring for patients with spinal disorders. These focus groups were recorded with written notes taken in addition. The data from these focus groups were divided into the key themes that were recurring with quotes from participants supporting the themes.

**FIGURE 3:**
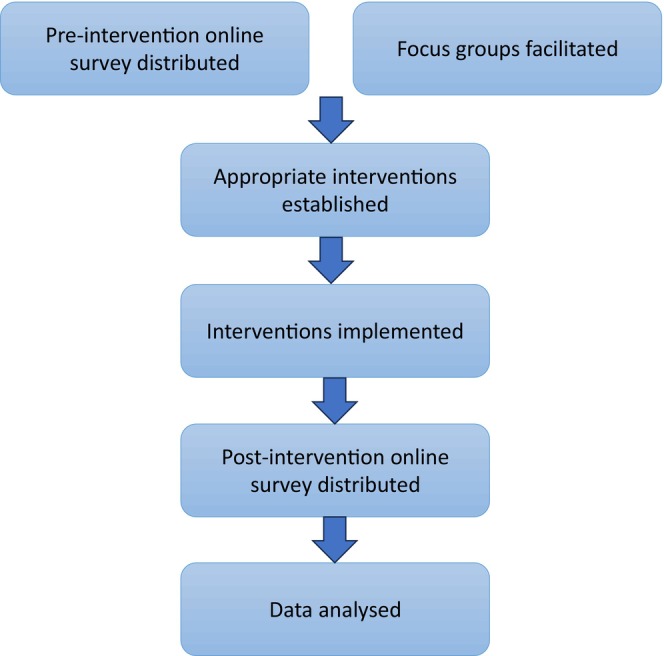
Data collection process.

In order to establish how successful the interventions had been in improving the confidence and competence of staff caring for patients with spinal disorders a post‐implementation online survey (Data S2) was then developed by the project team. This survey was distributed to all staff members who had accessed the e‐learning training package. This survey included both quantitative and qualitative questioning. Figure 3 demonstrates the data collection process chronologically.

#### 3.3.1 Ethics

This project was presented to the hospital ethics board and determined to be a service evaluation project with the trusts Research & Development Team. Thus, no further ethical approval was required. This was considered the most appropriate methodology as this process systematically assesses the implementation and impact of a project (NHS Institute for Innovation and Improvement, 2023). The results of this evaluation may then be transferable to others who are considering making the same or similar changes (NHS Institute for Innovation and Improvement, 2023). By utilizing the methodology of service evaluation and improvement, we can work towards making changes to improve patient outcomes, system performance and professional development (East London NHS Foundation Trust, 2023).

## 4 RESULTS

A total of 113 members of staff responded to the pre‐intervention online survey. Responses were received from all areas of the trust that routinely cared for adult patients with spinal disorders, with the majority of staff responding by working as part of the nursing teams (88%). Table 1 demonstrates the demographics of the survey respondents in terms of profession and speciality.

**TABLE 1:** Survey respondent demographics.

Speciality	Profession	Number of questionnaire responses pre‐intervention	*n* (%)
Critical care	Nurse	18	16
	Physiotherapist	5	4
Orthopaedics	Nurse	17	15
	Physiotherapist	1	1
	Occupational therapist	1	1
	Nursing assistant	2	2
Neurosciences	Nurse	15	13
	Physiotherapist	3	3
	Nursing assistant	2	2
Trauma	Nurse	15	13
	Physiotherapist	4	4
	Therapy assistant	1	1
Theatres	Nurse	6	5
	Operating department practitioner	1	1
Specialist surgery inpatients	Nurse	7	6
Neuro intensive care	Nurse	2	2
	Physiotherapist	2	2
Emergency department	Nurse	3	3
Other	Nurse	5	4
	Physiotherapist	1	1
	Therapy assistant	1	1
	Osteopath	1	1

### 4.1 Pre‐intervention data

Results from the online survey (Table 2) identified a lack of confidence and possible competence in assessing motor power of patients with spinal disorders and a need for training to be made available to staff. Seventy‐six of the 113 respondents (67%) had not received any formal training on how to complete a spinal motor assessment and 57% of respondents did not feel confident in completing a spinal motor assessment. Ninety per cent of staff responding to the questionnaire felt that training in spinal motor assessment would enhance their ability to care for patients with spinal disorders. Education/training was the most cited factor to increase confidence in spinal motor assessment (76%), and the second most cited factor was increased exposure to patients with spinal disorders (12%).

**TABLE 2:** Pre‐intervention survey results.

	Yes	No	Did not respond				
Have you had any formal training on SMA?	36 (32%)	76 (67%)	1 (1%)				
	Within clinical setting	Classroom	Online	Theory	N/A	Did not respond	
How was this training delivered?	28 (25%)	21 (19%)	4 (4%)	4 (4%)	21 (19%)	46 (41%)	
	Within the last 6 months	Within the last year	Within the last 3 years	Over 3 years ago	Did not respond		
When did you most recently have training?	4 (4%)	9 (8%)	14 (12%)	36 (32%)	50 (44%)		
	At least once a day	At least once a week	At least once a month	Less often than once a month	Never	Did not respond	
How often do you carry out SMA at work?	20 (18%)	10 (9%)	23 (20%)	24 (21%)	30 (27%)	6 (5%)	
	Extremely confident	Somewhat confident	Somewhat not confident	Not at all confident	Did not respond		
How confident do you feel undertaking a SMA?	6 (5%)	41 (36%)	23 (20%)	42 (37%)	1 (1%)		
	Extremely confident	Somewhat confident	Somewhat not confident	Not at all confident	Did not respond		
How confident so you feel caring for spinal surgery patients pre‐operatively?	14 (12%)	58 (51%)	24 (21%)	15 (13%)	2 (2%)		
	Extremely confident	Somewhat confident	Somewhat not confident	Not at all confident	Did not respond		
How confident do you feel caring for spinal surgery patients post‐operatively?	15 (13%)	65 (58%)	18 (16%)	13 (12%)	2 (2%)		
	Yes	No	Did not respond				
Do you feel training on SMA will enhance your ability to care for spinal surgery patients?	102 (90%)	9 (8%)	2 (2%)				
	Face to face	Group teaching	1:1 supervision and training	Online training	Other	Did not respond	
How do you feel this training would be best delivered?	82 (73%)	77 (68%)	40 (35%)	26 (23%)	4 (4%)	4 (4%)	
	Ward posters	Prompt cards	Electronic documentation templates	Electronic documentation prompts	Video refresher link	Other	Did not respond
What additional resources would be of benefit to further enhance your ability to complete a SMA?	64 (57%)	44 (40%)	65 (58%)	45 (40%)	66 (58%)	7 (6%)	6 (5%)
Themes from qualitative responses:	Education/training	Exposure/practice	Closer working relationship with spinal surgical teams	Resources	Documentation	Uniform assessment across professions	
What would increase your confidence in completing a SMA?	86 (76%)	13 (12%)	1 (1%)	2 (2%)	0 (0%)	1 (1%)	
What would increase your confidence in caring for a spinal surgery patient pre‐operatively?	52 (46%)	16 (14%)	9 (8%)	1 (1%)	4 (4%)	0 (0%)	
What would increase your confidence in caring for a spinal surgery patient post‐operatively?	57 (50%)	17 (15%)	7 (6%)	0 (0%)	2 (2%)	0 (0%)	

The focus group results (Table 3) were divided into themes with group consensus identified within each theme. Key points were as follows:

- Staff were uncertain on standard practice for completion of a spinal motor assessment and lacked confidence to complete this assessment.
- Staff were mostly completing an assessment of motor power but without awareness or understanding of myotomes, motor power grading and with no clear structure for documenting this.
- No formal training was available with the majority of learning picked up informally through observation of colleagues.
- It was felt that all staff members should access the same training to standardize practice across the trust.
- Staff were keen for an element of the training to be face to face with the opportunity to access further training to refresh knowledge and skills.
- Staff were keen for a closer working relationship with the spinal surgeons in order to improve communication around patient care.

**TABLE 3:** Focus group analysis.

Major themes	Most common responses (every focus group)	Common responses (Most focus groups or mentioned by multiple people)	Other responses (mentioned in 1 focus group, 1–2 persons only)	Group consensus/other notes
Initial thoughts and feelings on SMA	Most have a Good Theoretical understanding of what SMA entails—sensation + power + bladder/bowel + ASIA scoring. But lack confidence in practical execution Some senior nurses do regular spine motor assessments because you are experienced and aware but not for majority of staff and junior/newly qualified. At present, it is not a part of daily practice Large proportion of staff have not done a spinal assessment in UK. Knowledge comes from other sources, e.g., peers or epidural nursing training	For nurses who work in Neuro Wards Used to Brain Assessment, GCS, quick motor assessment But not used to asking spine‐specific questions, e.g., bladder/bowel.	Neglected Rare to get patient who has spinal injury without brain injury on this ward, so it makes the assessment difficult. Today I have a patient with both brain and spinal injury and unconscious. Our patients are not awake and alert. Also, our patients are confused so cannot participate in assessments as their responses are not reliable Changed Job—different practices in other cities compared to Oxford.	*Uncertain on standard practices in SMA* *Lack of confidence in performing SMA on their patients*.
Current SMA and documentation practices	Documentation lacking During transition from paper to EPR, patient handed over with empty spinal notes because it was not thought about during Assessment + Lack of confidence in Assessment Improvement in documents required Can be too much documentation options that do not need to be filled. Then, easy to miss those that do need to be. Things missed when documenting—not everything needs to be documented. Forgetting to record obs—especially agency staff Frequency of SMA use depends on which ward allocated/nurse speciality Not aware of documentation of spinal assessment and the correct scoring system	Have spinal surgery assessment (in EPR) as such but not a pop‐up so we do it if we have spinal pts we will do it frequently post‐op. Spinal Assessment does not form part of handover to Drs, unless nurse knows to do it because of experience. Recovery: Some need to know when to escalate change in power/sensation Follow surgeon post‐op instructions 1–1.5 h in recovery so some aspects are not possible, e.g., urinary output/retention. These need continued assessment when moved to ward; thus, handover needs to be done properly Perception that any limb movement equates to normal power, therefore, are just checking feet and toes. Has been incident where pt developed post‐op haematoma, slow to be identified. Assessment being done is basic, unsure if doing it correctly. Nothing to measure power against.	EPR documentation does not cover groin. Hard to see change. Spinal obs on SEND needed Doctors will not see a change on EPR Difficult to know how to document power. Not a space to document groin sensation. Limb obs are currently separate from other neuro obs Some have worked in the unit for over 5 years, but taken care of less than five spinal patients. Need for spinal assessment skills varies between departments/specialty. Good at the basics if they are doing enough Follow post‐op instructions SMA not established practice No knowledge of how to grade	*General consensus that nurses are doing some kind of assessment of motor function but lack of confidence that they are doing it correctly. Do not seem to be aware of myotomes. Not aware of MRC. Aware that their assessment is very subjective and are unsure as to how to document power*.
Confidence in managing spinal patients	Anxious in the beginning Feeling confident but not competent Needing more time to accurately assess spinal motor function Pain management Bladder/Bowel movements I have learnt spinal observation from my colleague. I still felt not competent enough to do observations After doing for few times felt confident	Confident and informed Empowered, More knowledge after training When assigned to a spinal patient, think about how the patient mobilizes, including need for assistance, log roll, pain management. Lack of complete knowledge Lack of information, practice Learn from practice daily After doing for few times felt confident Some felt confident because they are trained to do those assessments Limb power, definitely done for all pts but sensations we do not tend to do and feel less confident in doing it. Physio tends to do it Power: Depends on the person doing the assessment because it is subjective. Some find sensation easier.	Does the patient have any mobility restrictions? I Need more time to accurately assess spinal motor function Understands Pain management, Bowel movements Impression that spine patients are heavy Knows to make sure the spinal assessment section completed on EPR Mobility assessments Check power/sensation Pins and needles/Numbness Pain management Pt's frustration with repeat assessments Knowledge of: Pain level Mobility restrictions Bowel, bladder function Skin integrity Collar, braces, TLSO brace, JTO braces One area I freak out a bit is neurogenic bowel care because I do not think people understand the pathway so I think it is one area I think people need to be trained on.	
Thoughts on current training procedures	Very Ad Hoc, not much formal training Some happened to be trained by a consultant in the wards Or learnt from colleagues/seniors As a result, not confident in performing assessment alone Feel unsafe to perform assessment alone Need to see patients alongside colleagues, before building confidence to go solo.	Lack of patients to practise on No hands‐on showcase of, e.g., what is power 2,3 etc Reminders on paper/electronic forms, listing out specifically what needs to be done Need a quick summary reminder, rather than a long instruction sheet.	Lost from orientation programme Difficult to get space on orientation programme. No formal training—have had one off training from consultant which is now being disseminated from nurse to nurse. Informal training during supernumerary time. Has not been on orientation for at least 5 years. One person had additional learning from the neuro course One person had additional knowledge from previous role Most learnt from colleagues ‘on the job’ Need more training—myotomes, etc. Other training needs around spine—bladder/bowel	*No formal training in place. Nearly all learning was picked up informally ‘on the job’*.
Suggested areas for improvement for SMA training	A structured training programme Video guides to showcase clearly what is required. E‐learning is great, but hands‐on is imperative. Need to have one‐to‐one training with a patient for practice How to document observed results properly Drop down box will be useful How to properly assess power E.g., some patients might have strength, but pain inhibits them from certain movements. Some alternative movements to assess. Modes to retain information Senior nurses find that junior nurses forget how to perform the assessment, due to a lack of repetitive practice and familiarity Summary Card for reminders Groin Assessment—Bladder/Bowel Notes on Spinal Documents on how to assess Standardized documents on EPR Training on Management of Spinal Patients How to assist in moving them, log roll, collar	Renewal training/assessment every 2 years. One year too frequent. Summary cards and pointers on documentation, for refreshers to help nurses retain information Notes on Websites—easy access via staff computers Mix between preference for face‐to‐face versus Microsoft Teams Two parts—theory and demonstration Video that shows normal and abnormal, Video on learning hub Theory and practical with scenarios. How often should we be assessing patients?	SMA competencies Practice sessions Exam at the end yearly/2 yearly (2 = more achievable) Instant access on sharepoint/yammer could be a way to make it easily accessible. Doctor from spinal team involved in training Standardized training to ensure that all staff are doing the same. Feel e‐learning does not teach enough. Also, need to train senior nurses to be trainers. Same level of training for B5 & B6 Everyone needs to be able to access practical training. Identify 1:1 sessions with clin ed when appropriate patients admitted. Video Easy to assess Simulation Video that shows normal and abnormal Video on learning hub Theory and practical with scenarios. How often should we be assessing patients? Two parts, theory and demonstration	*Would need initial face to face training with some kind of update available. Should be some kind of assessment/competencies after initial training. Everyone should have the same training so all doing the same in terms of assessment*.
Final thoughts	Will be good to know the team Need to standardize care, and support nurses to look after their pts. Clear instructions on what they want and how they want it Spine Nurses love their job! They enjoy looking after spinal patients, provide different skills and variation to routine compared to neuro brain patients. E‐learning following main training to remind	There is inconsistency in many aspects that cannot be helped – e.g., how regularly the doctors see their pts, how often nurses should carry out observations, methods of assessment, how findings are documented Takes a while to navigate spinal documentation on EPR. Current EPR sometimes does not allow for easy documentation of certain pieces of information—fit in notes instead. Want for EPR to be more visible, flexible and need to make staff aware that it is there Individualized care—SOP	Poor communication from spine Slow to answer bleeps Do not communicate with nursing staff on ward round Sometimes no documentation until later in the day when morning shift is ending Ortho spine particularly hard to contact SAH pathway = perfect—fluids/laxatives. Needs a whole pathway review. General opinions—more used to head pts than completing SMA Trying to improve practice—adding prompts onto handover More engagement from spinal team—discuss patients with nursing staff and what they expect in terms of outcome (teamworking) Nursing Staff should be given classroom sessions with assessment on competencies prior to caring for spine patients. Proficiency assessments 1‐ to 3‐h study session on induction with digital backup run by experienced nurses/physios/doctors who understand what goes wrong Should be at least one assessment and updates if there are changes in how to care for these patients	*Would like an improved relationship with spine teams. Would like conversations with the team when patients are reviewed*. *Would like to work more closely with spinal surgeons*. *Training should include face to face with some kind of assessment. Digital training as refresher would be appropriate*.

Abbreviation: SMA, Spinal Motor Assessment.

### 4.2 Post‐intervention data

The post‐intervention questionnaire was distributed to all members of staff who had accessed the e‐learning training package. The majority of responses were from nursing staff (87%) with 92% of responses from clinicians working within either neurosurgery, orthopaedics or trauma. The results of the online survey were collated (Table 4).

**TABLE 4:** Post‐intervention survey results.

	Yes	No			
Have you completed the SMA e‐learning training package?	72 (97%)	2 (3%)			
	Classroom	Within clinical setting	Online training through ASIA	Other online training	Other
If you have had training on how to complete a SMA, in addition to the e‐learning training package, in what format was this?	31 (42%)	28 (38%)	5 (7%)	3 (4%)	7 (9%)
	Fully	To a great extent	To some extent	Minimally	Not at all
Do you feel the SMA e‐learning package has increased your knowledge and understanding of SMA?	13 (18%)	43 (58%)	11 (15%)	7 (9%)	0 (0%)
	Fully	To a great extent	To some extent	Minimally	Not at all
Do you feel the SMA e‐learning package has increased your competence in undertaking a SMA?	11 (15%)	42 (57%)	14 (19%)	7 (9%)	0 (0%)
	Extremely confident	Somewhat confident	Somewhat not confident	Extremely not confident	
How confident do you feel undertaking a SMA after the training?	16 (22%) (17% increase)	53 (72%) (36% increase)	5 (7%) (13% decrease)	0 (0%) (37% decrease)	
	Yes	No	Other		
Has your confidence increased since completing the training?	67 (91%)	5 (7%)	2 (3%)		
	Extremely confident	Somewhat confident	Somewhat not confident	Not at all confident	Did not answer
How confident do you feel caring for spinal surgery patients pre‐operatively?	19 (26%) (14% increase)	50 (68%) (17% increase)	3 (4%) (17% decrease)	1 (1%) (12% decrease)	1 (1%)
	Extremely confident	Somewhat confident	Somewhat not confident	Not at all confident	
How confident do you feel caring for spinal surgery patients post‐operatively?	18 (24%) (11% increase)	54 (73%) (15% increase)	2 (3%) (13% decrease)	0 (0%) (12% decrease)	
	Posters	Prompt card	Video refresher	Spinal motor assessment guide	
Of the additional resources provided to support you, which have you accessed?	30 (41%)	31 (42%)	24 (32%)	50 (68%)	
	Extremely useful	Somewhat useful	Somewhat not useful	Not at all useful	
Overall, how useful have you found the additional resources?	35 (47%)	37 (50%)	1 (1%)	1 (1%)	

Themes from qualitative responses:	Increased understanding/more confident	Revision/felt confident prior to training	Unspecific positive response to training	Increased understanding but further learning required	Other	
Having undergone the training, how has your understanding of SMA changed?	49 (66%)	10 (14%)	8 (11%)	3 (4%)	3 (4%)	
	Improving understanding of assessment/assessment technique/increased confidence	Improved efficiency in identifying or escalating deterioration/improvement in patient safety or quality of care	Improved education tool	Anticipate time challenges to complete assessment	Improved documentation	Other
Please give details on if/how this will influence your practice	51 (69%)	17 (23%)	3 (4%)	2 (3%)	1 (1%)	8 (11%)
	Resources deemed to be helpful to support assessment completion	Easily accessible	Useful for teaching	Not seen any		
Please give further details on the usefulness of the additional resources	62 (84%)	3 (4%)	2 (3%)	1 (1%)		
	Ongoing training or support to further enhance knowledge and skills	More easy to access visual reminders	Experience with patients	Increased managerial support/ensure all staff groups complete training	Clear post‐op protocol for spinal motor assessment	Nil further to be done
What could be done in addition to this project to further improve the completion of Spinal Motor Assessment across the trust?	24 (32%)	5 (7%)	3 (4%)	3 (4%)	1 (1%)	3 (4%)

At the time of data collection, 275 members of staff, not affiliated with implementing the project, had accessed the e‐learning training package with 208 members of staff having completed and passed the e‐assessment. Seventy‐four members of staff responded to the survey, we can, therefore, calculate a response rate of 27%.

The data demonstrate the overall positive impact of the project on clinician's confidence and competence in completing a spinal motor assessment with 100% of respondents reporting increased competence and 94% of respondents reporting feeling confident to complete the assessment (a 53% increase compared to pre‐intervention data). The data also demonstrate an overall increase in confidence caring for spinal patients when compared with preintervention data with 31% more respondents feeling confident to care for spinal surgery patients preoperatively and 26% more respondents feeling confident caring for spinal surgery patients post‐operatively. The data also indicate that additional resources were felt to be of benefit to clinicians with 97% of respondents reporting they found them useful.

The qualitative findings from the open‐ended questions in the postintervention online survey (Data S2) reinforce the quantitative data from the study with respondents reporting feeling *‘a lot more confident in assessing patients who have undergone spinal surgery’*. When asked how their understanding of Spinal Motor Assessment had changed respondents reported *‘understanding had changed tremendously’* with a *‘better understanding of why we do Spinal Motor Assessment and escalate changes’*. Respondents reported that this learning would change their practice with clinicians able to *‘complete the assessment with the correct knowledge of the process’* and with *‘more confidence in assessing (spinal patients)’* as well as ‘*a greater understanding of the underlying issues…..and the implications….findings have’*. Respondents identified that this learning would help staff to ‘*detect early warning signs if there is any deterioration of the patient’* and for *‘patients in our care to be treated in a timely manner’*. Other positive outcomes identified by the respondents were that they were able to complete a *‘more accurate assessment’*, had *‘confidence in……supporting staff members’*, were *‘able to share the knowledge with colleagues’* had become *‘more attentive to neurological change’* and could *‘act on it’* with an overall sense that the changes in practice *‘improves quality of care’*.

## 5 DISCUSSION

This study aimed to understand and address the challenges around the completion of spinal motor assessment across the trust in order to enhance the safety of patients with spinal disorders. The data demonstrated that the challenges mostly stemmed from a lack of targeted training. The project team were able to address this through provision of education, additional resources and a digital documentation template.

### 5.1 Training

Pre‐intervention survey results demonstrate the low levels of self‐reported confidence and competence in caring for this patient group across the trust, thereby identifying a gap in the training provision. Digital education is ‘the act of teaching and learning by means of digital technologies’ (Car et al., 2019). Digital education is increasingly being used within healthcare to provide training to staff groups (Martinengo et al., 2019) in a way that allows flexibility of access, the ability to reach large numbers of staff members and ensure standardized content is taught across the cohort whilst mitigating factors such as workload, distance from learning centres or costs (Du et al., 2013; Shahhosseini & Hamzehgardeshi, 2015). Within this project, digital education was identified as being a solution to provide training to a large number of clinicians in a time‐efficient manner. By utilizing a digital training platform, the longevity of access to this training can be maintained whilst remaining cost neutral to the NHS. The use of digital education allowed funding to be utilized to create resources to support clinicians with assessment completion as well as providing additional face‐to‐face ‘Train the Trainer’ training to condense knowledge and understanding and to refine assessment technique. Within this project, the quantitative data strongly demonstrate that digital learning has had an impact on staff practices within the trust, when caring for spinal patients, with 91% of survey respondents reporting that the e‐learning training package has influenced their practice. This is in keeping with the evidence that supports the effectiveness of digital learning as a means of providing education within a healthcare setting (Du et al., 2013; Martinengo et al., 2019; Shahhosseini & Hamzehgardeshi, 2015) with studies demonstrating that digital education for nursing knowledge acquisition and retention has produced superior, or at least the same effects to traditional methods (Du et al., 2013).

### 5.2 Digital assessment tools/documentation

The concept of digital assessment and documentation to prompt staff to escalate concerns was utilized in this project through adapting the existing spinal surgery assessment tool within the electronic patient records. This updated tool prompts a standardized patient assessment, allowing for documentation of baseline motor function and through regular assessment empowers staff to identify early and clearly document any signs of deterioration in a way that trends could be easily identified for escalation. Through documenting this digitally information can be viewed remotely by appropriate professionals as part of the escalation process. This assessment and documentation process has now become standard practice throughout the trust allowing for a consistent escalation process and essential clinical information being available for the responsible team to review and act upon appropriately. Digital documentation is increasingly becoming the norm in healthcare settings with the NHS Long‐Term Plan including the ‘ability for clinicians to access and interact with patient records and care plans wherever they are’ as one of the practical priorities in NHS digital transformation (NHS, 2019). These digital technologies in healthcare can provide the ability for templates, digital alerts and prompts to be utilized as well as allowing remote review of patient records. This combination is more time efficient for healthcare professionals and when used optimally can promote in‐depth patient assessment. The use of digital assessment and documentation prompting staff to escalate concerns was recommended as far back as 1999 by the Audit Commission (Gardner‐Thorpe et al., 2006) in relation to early warning systems. Since it is original development, this has gone through modifications resulting in the current National Early Warning System 2 (NEWS2) (Williams, 2022) which is used throughout the NHS. The purpose of these systems is to facilitate prompt communication between nursing staff/allied health professionals and medical staff when deterioration first becomes apparent (Gardner‐Thorpe et al., 2006).

### 5.3 Financial implications

Machin et al. (2018) state that between April 2012 and April 2017, clinical negligence claims in spinal surgery were estimated to cost the NHS £535.5 million with the most common cause for claims being judgement/timing. Although the drive for this project was patient safety and enhanced care, we should not ignore the potential financial benefits through mitigating risk of litigation claims, thereby potentially reducing the trusts medicolegal costs along with the stress and anxiety these processes can cause to staff members.

### 5.4 Challenges

Within this trust patients with spinal disorders can be admitted via a number of pathways, to a number of ward areas, under one of two key surgical teams (Figure 1) with differing education and governance processes. The challenges this creates for staff are reflected by themes within the qualitative data whereby staff, unprompted, raised the desire for a closer working relationship with the spinal surgery teams and identify their uncertainty on standard practice for this patient group as well as the need for more exposure to this patient group. Along with this partially being the drive for the project, this was also a factor in resistance to the changes being implemented.

Resistance to change is defined as a behaviour aimed at impeding or ceasing change (DuBose & Mayo, 2020). Individuals behaviours may be influenced by mistrust, fear of change and communication barriers (DuBose & Mayo, 2020). The project team mitigated this through ongoing stakeholder involvement to support staff understanding of the need for change and to allow staff a forum to contribute to implementation of change. As indicated by the demographics of the respondents to the post‐implementation questionnaire, there was more engagement from some areas of the trust than others. This can be attributed to higher levels of managerial support towards the project from the point of initiation in these areas with staff allocated time to access training. Areas that demonstrated less engagement in accessing training reported to the project team that patients with spinal disorders, as a percentage of their patient group, were lower than that of other conditions and thus gave priority to other staff training requirements. The production of an online training programme negates the requirement for a time frame for staff to access this; therefore, delayed engagement from staff groups does not restrict them from learning. However, ultimately, we can anticipate that staff groups are more likely to prioritize increasing their skills, competence and confidence in caring for patients with spinal disorders if this patient group make up a more substantial number of their total caseload, which could be achieved in this trust through co‐locating patients into fewer clinical areas.

## 6 LIMITATIONS

This report utilized staff self‐reported outcomes pre‐ and post‐intervention. The compliance with the changes in practice of completing regular spinal motor assessment of patients with spinal disorders and the impact on patient safety has not been formally assessed. This could be done by retrospectively reviewing patient records to document the pre‐intervention standard of neurological observation compared with that post‐intervention.

A limitation of the results is the questionnaire response rate. The pre‐intervention questionnaire was distributed throughout the trust via corporate communications and divisional leads; therefore, without a definite number of staff that this was shared with we are unable to calculate a response rate. The post‐intervention response rate was 27%. Although this response rate would be considered low, when we look at the number of responses, 74, and the consistently overall positive responses to the survey the project team felt that this was high enough for us to draw the conclusions detailed in this report.

## 7 CONCLUSION

This study highlights the importance of targeted education to ensure clinicians are appropriately skilled to identify neurological deterioration in patients with spinal disorders. Through engaging with clinicians to establish and address educational needs, and through utilization of digital education, this quality improvement project has successfully increased competence and confidence in this area of spinal care. The overall outcome is an environment whereby patient safety is enhanced through reducing the risk of delayed identification of neurological deterioration.

## AUTHOR CONTRIBUTIONS

JB, ED, BB, LS, GM: Made substantial contributions to conception and design, or acquisition of data or analysis and interpretation of data; JB, LS, GM: Involved in drafting the manuscript or revising it critically for important intellectual content; JB, ED, BB, LS, GM: Given final approval of the version to be published. Each author should have participated sufficiently in the work to take public responsibility for appropriate portions of the content; JB, GM: Agreed to be accountable for all aspects of the work in ensuring that questions related to the accuracy or integrity of any part of the work are appropriately investigated and resolved.

## FUNDING INFORMATION

We would like to thank the Burdett Trust for Nursing for providing funding for this project.

## CONFLICT OF INTEREST STATEMENT

None declared by the authors.

## PEER REVIEW

The peer review history for this article is available at https://www.webofscience.com/api/gateway/wos/peer‐review/10.1111/jan.16399.

## PRESENTED AT

This work was presented as a poster presentation at the International Forum on Quality and Safety in Healthcare, Copenhagen, 2023 and as an oral presentation at the British Association of Spine Surgeons Conference, Bournemouth, 2024.

## Supporting information


Data S1.

Data S2.

Data S3.

## Data Availability

The data that supports the findings of this study are available in the supplementary material of this article.
