# Supplementary material for: Safety in spinal surgery—Empowering clinicians to report concerns in motor function
Source: J Adv Nurs. 2024 Aug 20;81(9):5546–62. doi: 10.1111/jan.16399 (PMC12371831; doi:10.1111/jan.16399)
Supplement: Supplementary file 1 — Data S1. [file JAN-81-5546-s001.pdf]

Caption: Pre-Intervention Survey

Spinal Motor Assessment

The survey will take approximately 4 minutes to complete.
Thank you for taking the time to complete this questionnaire.
This questionnaire has been designed to help us understand the current training needs within Oxford University Hospitals towards completing Spinal Motor Assessments.
Please give as much detail as possible in the free text boxes as this will help guide our training programme and may lead to future quality improvement projects.
Please note this questionnaire is anonymous.

1. What is your profession?

- ◯ Doctor
- ◯ Nurse
- ◯ Physiotherapist
- ◯ Occupational therapist
- ◯ Speech and language therapist
- ◯ Other

2. Within which speciality do you work?

- ◯ Neurosciences
- ◯ Trauma
- ◯ NICU
- ◯ Critical care
- ◯ Emergency department
- ◯ Theatres
- ◯ Paediatrics
- ◯ Other

3. What is your understanding of a spinal motor assessment?

4. Have you had any formal training on spinal motor assessment?

- ◯ Yes
- ◯ No

5. How was this training delivered?

- [ ] Within clinical setting
- [ ] Classroom
- [ ] Online
- [ ] Theory
- [ ] Other

6. When did you most recently have training?

- ○ Within the last 6 months
- ○ Within the last year
- ○ Within the last 3 years
- ○ Over 3 years ago

7. How often do you carry out spinal motor assessments at work?

- ○ At least once a day
- ○ At least once a week
- ○ At least once a month
- ○ Less often than once a month
- ○ Never

8. How confident do you feel undertaking a spinal motor assessment?

- ◯ Extremely confident
- ◯ Somewhat confident
- ◯ Somewhat not confident
- ◯ Not at all confident

9. What would increase your confidence?

10. How confident do you feel caring for a spinal surgical patient pre-operatively?

- ◯ Extremely confident
- ◯ Somewhat confident
- ◯ Somewhat not confident
- ◯ Not at all confident

11. What would increase your confidence?

12. How confident do you feel caring for a spinal surgical patient post-operatively?

- ◯ Extremely confident
- ◯ Somewhat confident
- ◯ Somewhat not confident
- ◯ Not at all confident

13. What would increase your confidence?

14. Do you feel training in spinal motor assessment would enhance your ability to care for spinal surgery patients?

- ◯ Yes
- ◯ No

15. How do you feel this training would be best delivered?

- [ ] Face to face
- [ ] Group teaching
- [ ] 1:1 supervision and training
- [ ] Online training
- [ ] Other

16. What additional resources would be of benefit to further enhance your ability to complete a motor assessment of a spinal patients?

- [ ] Ward posters
- [ ] Credit card sized prompt cards
- [ ] EPR templates
- [ ] EPR prompts
- [ ] Video refresher link
- [ ] Other

17. Any other comments?

This content is neither created nor endorsed by Microsoft. The data you submit will be sent to the form owner.

Microsoft Forms
