# Supplementary material for: Safety in spinal surgery—Empowering clinicians to report concerns in motor function
Source: J Adv Nurs. 2024 Aug 20;81(9):5546–62. doi: 10.1111/jan.16399 (PMC12371831; doi:10.1111/jan.16399)
Supplement: Supplementary file 2 — Data S2. [file JAN-81-5546-s003.pdf]

Caption: Post-intervention Survey

Post Implementation Questionnaire - Spinal Motor Assessment Training

The survey will take approximately 4 minutes to complete.

Thank you for taking the time to complete this questionnaire.
This questionnaire has been designed to give staff the opportunity to feedback on the Spinal Motor Assessment training. Your input is invaluable in measuring the success of the project, identifying areas for further improvement and guiding how best to implement future quality improvement projects.

Please note this questionnaire is anonymous.

* Required

1. What is your profession? *

- ◯ Nurse
- ◯ Physiotherapist
- ◯ Occupational therapist
- ◯ Other

2. Within which speciality do you work? *

- ◯ Neurosciences
- ◯ Trauma
- ◯ Oxford Critical Care Unit
- ◯ Orthopaedics
- ◯ Paediatrics
- ◯ Emergency Department
- ◯ Theatres
- ◯ NICU
- ◯ Other

3. Have you completed the Spinal Motor Assessment training package on My Learning Hub? *

- ◯ Yes
- ◯ No

4. If you have had training on how to complete a Spinal Motor Assessment, in addition to the training on My Learning Hub, in what format was this? *

- ◯ Classroom
- ◯ Within clinical setting
- ◯ Online training through American Spinal Injuries Association (ASIA)
- ◯ Other online training
- ◯ Other

5. Having undergone the training, how has your understanding of Spinal Motor Assessment changed? *

6. Do you feel the Spinal Motor Assessment e-Learning Training Package on My Learning Hub will influence your practice? *

- ◯ To a great extent
- ◯ To some extent
- ◯ Minimally
- ◯ Not at all

7. Please give details on if / how this will influence your practice. *

8. Do you feel the Spinal Motor Assessment Training Package on My Learning Hub has increased your knowledge and understanding of Spinal Motor Assessment? *

- ○ Fully
- ○ To a great extent
- ○ To some extent
- ○ Minimally
- ○ Not at all

9. Do you feel the Spinal Motor Assessment Training Pack on My Learning Hub has increased your competence in undertaking a Spinal Motor Assessment? *

- ○ Fully
- ○ To a great extent
- ○ To some extent
- ○ Minimally
- ○ Not at all

10. How confident do you feel undertaking a Spinal Motor Assessment after the training *

- ◯ Extremely confident
- ◯ Somewhat confident
- ◯ Somewhat not confident
- ◯ Extremely not confident

11. Has your confidence increased since completing the training? *

- ◯ Yes
- ◯ No
- ◯ Other

12. How confident do you feel caring for spinal surgery patients pre-operatively?

- ◯ Extremely confident
- ◯ Somewhat confident
- ◯ Somewhat not confident
- ◯ Not at all confident

13. How confident do you feel caring for spinal surgery patients post-operatively?

- ◯ Extremely confident
- ◯ Somewhat confident
- ◯ Somewhat not confident
- ◯ Not at all confident

14. Of the additional resources provided to support you, which have you accessed? *

- ☐ Posters
- ☐ Prompt card
- ☐ YouTube link to video refresher
- ☐ Spinal Motor Assessment Guide

15. Overall how useful have you found the additional resources? *

- ◯ Extremely useful
- ◯ Somewhat useful
- ◯ Somewhat not useful
- ◯ Not at all useful

16. Please give further details on the usefulness of each of the additional resources. *

17. What could be done in addition to this project in order to further improve the completion of Spinal Motor Assessment across the trust?

This content is neither created nor endorsed by Microsoft. The data you submit will be sent to the form owner.

Microsoft Forms
